# Prophylactic and curative effect of rosemary leaves extract in a bleomycin model of pulmonary fibrosis

**DOI:** 10.1080/13880209.2016.1247881

**Published:** 2017-01-16

**Authors:** Sana Bahri, Ridha Ben Ali, Khaoula Gasmi, Mona Mlika, Saloua Fazaa, Riadh Ksouri, Raja Serairi, Saloua Jameleddine, Vadim Shlyonsky

**Affiliations:** a Laboratory of Physiology, Faculty of Medicine of Tunis, University of Tunis El Manar, Tunis, Tunisia;; b Laboratory of Physiopathology and Pharmacology, Faculty of Medicine, Université Libre de Bruxelles, Brussels, Belgium;; c Laboratory of Experimental Medicine, Faculty of Medicine of Tunis, University of Tunis El Manar, Tunis, Tunisia;; d Laboratory of Anatomy and Pathology, Abderhaman Mami Hospital, Ariana, Tunisia;; e Laboratory of Physiology, Faculty of Science of Tunis, University of Tunis El Manar, Tunis, Tunisia;; f Laboratory of Eco-Process and Valorization of Aromatic and Medicinal Plants, Center for Biotechnology, Technopole Borj Cédria (CBBC), Tunis, Tunisia;; g High School of Health Sciences, Tunis, Tunisia;

**Keywords:** Polyphenols, high-performance liquid chromatography, antioxidant, antifibrotic

## Abstract

**Context:** Pulmonary fibrosis is a devastating disease without effective treatment. Rosemary is appreciated since ancient times for its medicinal properties, while biomolecules originated from the plant have an antioxidant and antifibrotic effect.

**Objective:** The effects of *Rosmarinus officinalis* L. (Lamiaceae) leaves extract (RO) on bleomycin-induced lung fibrosis were investigated.

**Materials and methods:** Male Wistar rats were given a single dose of bleomycin (BLM, 4 mg/kg, intratracheal), while RO (75 mg/kg, intraperitoneal) was administered 3 days later and continued for 4 weeks (BLM/RO1-curative group). Alternatively, RO was administered 2 weeks before BLM and continued 15 days thereafter (BLM/RO2-prophylactic group). Antioxidant activities of RO and lung tissues were studied by standard methods. Histological staining revealed lung architecture and collagen deposition. RO was characterized for its polyphenol content and by high-performance liquid chromatography.

**Results:** RO polyphenol content was 60.52 mg/g of dry weight, carnosic and rosmarinic acids being major components (6.886 and 2.351 mg/g). Antioxidant effect of RO (DPPH and FRAP assay) expressed as IC_50_ values were 2.23 μg/mL and 0.074 μg/mL, respectively. In BLM/RO1 and BLM/RO2 lung architecture was less compromised compared to BLM, which was reflected in lower fibrosis score (2.33 ± 0.33 and 1.8 ± 0.32 vs 3.7 ± 0.3). Malondialdehyde levels were attenuated (141% and 108% vs 258% of normal value). Catalase and glutathione-*S*-transferase activities were normalized (103% and 117% vs 59%, 85% and 69% vs 23%, respectively).

**Discussion and conclusion:** RO has a protective effect against BLM-induced oxidative stress and lung fibrosis due to its phenolic compounds.

## Introduction

Idiopathic pulmonary fibrosis (IPF) is the most serious form of interstitial pneumonia. This disease of unknown cause affects a population between 50 and 70 years with a median survival after diagnosis of only 3–5 years. The physiopathological mechanisms behind the fibrotic process and architectural disorganization are still imperfectly understood (Cottin 2002; Crestani et al. 2005; Cottin & Cordier 2007). The long prevailing concept, which was that of chronic inflammation leading to fibrosis is controversial (Gauldie 2003), but there has been a shift in the understanding of the physiopathology of IPF from that of a chronic inflammatory state to abnormal wound healing. This concept is based on the important interaction between alveolar epithelium and lung fibroblasts more than on alveolar inflammation.

Many therapeutic clinical trials were performed to treat this devastating disease, but the efficacy of current treatments, based on the association of corticosteroids and immunosuppressive factors, remains questionable because they have not shown an improvement in patient survival. Currently, lung transplantation is still the only efficient treatment, but despite its success, the risks of complications are possible including infections owing to immunosuppression and the rejection of acute and chronic transplant (Fioret et al. 2011). Consequently, the management of IPF needs to investigate the effect of new molecules with an acceptable tolerance profile in clinical trials.

*Rosmarinus officinalis* L., originally from the Mediterranean basin, belongs to the Lamiaceae family, which comprises up to 200 genera and about 3500 species (Héthelyi et al. 1987). It is an aromatic plant with an intense pleasant smell, green leaves and a flowering season running from April to August. *Rosmarinus officinalis* is one of the most widely commercialized plants used as a culinary herb for flavouring and as an antioxidant in processed foods and cosmetics (Zheng & Wang 2001). *Rosmarinus officinalis* is also exploited for its essential oil and its richness in polyphenols (Rozman & Jersèk 2009), and is widely used in folk medicine, cosmetics and phytocosmetics (Pintore et al. 2002). Several rosemary extracts from dried leaves have been described to have antioxidant (Aruoma et al. 1996), anti-inflammatory and anticancer activities (Peng et al. 2007). Most of these observed effects are related to the phytochemical compounds of this herb obtained through several extraction methods, mainly carnosic and rosmarinic acids (Afonso & Sant’AnaL 2008; Pérez-Fons et al. 2010).

Carnosic acid is the main phenolic diterpene compound reported to have chemopreventive (Nabekura et al. 2010), antioxidant, antimicrobial (Bernardes et al. 2010), anti-obesity, antiplatelet (Kelsey et al. 2010) and antitumor activities (Yesil-Celiktas et al. 2010).

Rosmarinic acid has a number of biological properties, such as antioxidant (Shanlou et al. 2005; Sui et al. 2012), anti-inflammatory (Osakabe et al. 2004), antiangiogenic (Huang & Zheng 2006) anti-apoptotic (Kim et al. 2005), antifibrotic (Li et al. 2010) chemoprotective (Debersac et al. 2001), neuroprotective (Fallarini et al. 2009), the photoprotection of keratinocytes (Psotova et al. 2006) and the prevention of Alzheimer's disease (Hamaguchi et al. 2009). *Rosmarinus officinalis* also contains other derivatives such as ursolic acid, oleanolic acid and micromeric acid, which are reported to have anti-inflammatory and antitumor activities (Mengoni et al. 2011).

The presence of phenolic diterpenes, flavonoids, tannins and phenolic acids in plants make them a potential source of natural antioxidants (Dawidowicz et al. 2006). Therefore, in this study, we analyzed the effects of rosemary leaves extract in a model of experimental pulmonary fibrosis induced by bleomycin in rats. We propose that at least, in part, *Rosmarinus officinalis* can exert an antioxidant and antifibrotic effect due to the presence of several bioactive compounds.

## Materials and methods

### Plant sampling

The aerial parts of *Rosmarinus officinalis* (100 g) were collected by Dr. Raja Serairi (High School of Health Sciences, Tunis, Tunisia) during the flowering period in July 2013 from the mountains of Boukornine (36°41′22*″* North latitude and 10°21′25*″* East longitude) located in northeastern Tunisia. The botanical identification of *R. officinalis* was performed by Professor Riadh Ksouri (Biotechnology Center in Borj-Cedria, Tunisia) and a voucher specimen has been deposited in the Biotechnology Center in Borj-Cedria (R-RV 269).

### Extract preparation for biological activities

*Rosmarinus officinalis* leaves were air dried in shade at ambient temperature and crushed to obtain a fine powder. We used Soxhlet apparatus for the extraction and ethanol (70%) as a solvent. Extracts were kept for 24 h at 4 °C, filtered, evaporated at 35 °C with a rotary vacuum evaporator and finally lyophilized. The obtained powder was resuspended in physiologic serum before testing.

### Determination of phenol compound contents

Colorimetric method was used to determine total polyphenol and flavonoids content, as described by Dewanto et al. (2002). Total condensed tannins were quantified as described by Sun et al. (1998). All samples were analyzed in triplicate.

### Antioxidant activities of Rosmarinus officinalis: DPPH assay

DPPH (2,2-diphenyl-1-picrylhydrazyl) quenching capacity of plant extracts was determined according to Hanato et al. (1988). Briefly, we incubated 1 mL of the Soxhlet samples with 250 μL of 0.2 mM solution of DPPH for 30 min at ambient temperature, then we read the absorbance against a blank at 517 nm. The percentage of inhibition of DPPH free radical (I %) was calculated as follows:

$$I\left(\%\right)=\left[\left(A_{0}-A_{1}\right)/A_{0}\right]\times 100.$$

where A_0_ is the absorbance of the control (BHT: butylated hydroxytoluene) and A_1_ is the absorbance of the sample at 30 min. All samples were analyzed in triplicate and the results are expressed as IC_50_ (μg/mL) corresponding to the inhibiting concentration of 50% of the synthetic radical.

### Antioxidant activities of *Rosmarinus officinalis*: iron reducing power (FRAP assay)

The ability of plant extracts to reduce Fe^3+^ was determined exactly as described by Oyaizu (1986). The method is based on the incubation of samples in sodium phosphate buffer (0.2 M, pH = 6.6) with potassium ferricyanide (1%), followed by the addition of the trichloroacetic acid (10%) and centrifugation at 650*g* for 10 min. The supernatant was then mixed with the ferric chloride solution in water and the absorbance was measured at 700 nm. Ascorbic acid was used as a positive control. Results are expressed as an EC_50_ value (mg/mL), which is the effective concentration giving an absorbance corresponding to a 0.5 for reducing power and which is obtained from linear regression analysis.

### HPLC analysis

In order to separate and identify the compounds from the rosemary ethanol extract, reversed phase high-performance liquid chromatography (Agilent Technologies 1260, Munich, Germany) equipped with an UV diode array detector (DAD) and C18 column (4.6 mm ×100 mm) packed with 3.5 μm diameter particle was carried out.

The mobile phase was a mixture of two solvent compositions, that of solvent A (ethanol) and solvent B (milliQ water containing 0.1% formic acid), and the composition gradient was 10% of A up to 5 min and was amended to obtain 20, 30, 50, 70, 90, 50 and 10% at 5, 10, 15, 20, 25, 30 and 35 min, respectively. The column temperature was maintained at 25 °C, the volume injected was 2 μL and the flow rate was set at 0.4 mL/min.

Detection of compounds in HPLC fractions was adapted from Albu et al. (2004) because in this study wavelength of 284 nm was found to be optimal for the detection of rosmarinic and carnosic acids. These compounds also have a good absorbance at 280 and 254 nm, and these wavelengths were chosen in this experiment for analysis.

The chromatography peaks have been identified following the comparison of their retention time with those of reference standards injected under the same chromatographic conditions and monitored with DAD, and spectra were recorded between 200 and 400 nm. The quantification of each compound was based on the calibration curve of the corresponding standard rosmarinic acid: *Y* = 7.933*x* – 18.96 (*r* = 0.999) and carnosic acid: *Y* = 1.400*x* + 27.37 (*r* = 0.997).

### Animal model of bleomycin-induced lung fibrosis

Fifty Wistar adult male rats (Pasteur Institute, Tunis, Tunisia), weighing between 180 and 220 g were supplied with food and water *ad libitum* and maintained in animal housing at a controlled temperature (22 ± 2 °C) with a 12 h light–dark cycle. All experiments were performed according to the recommendations of the ethic committee of Tunis University for care and use of animals in conformity with NIH guideline.

Bleomycin rat model of lung fibrosis as reported earlier by Serairi Beji et al. (2014) was used. All rats underwent anaesthesia by intraperitoneal injection of 100 μg/g of pentobarbital sodium solution (Sandoz Laboratory, Paris, France). Each anaesthetized rat was immediately fixed on a gallows. Induction of fibrosis was done by an intra-tracheal injection of 4 mg/kg body weight (bw) of bleomycin sulfate solution (Bleomycin^®^, Laboratories Aventis, Paris, France).

### Experimental design

Rats were divided into five groups of 10 animals each:

Group I received a single intra-tracheal injection of normal saline (control group).

Group II received a single intratracheal instillation of bleomycin (4 mg/kg bw) (rat model of lung fibrosis: BLM group).

Group III received a daily intraperitoneal injection of RO (75 mg/kg bw) for 4 weeks (RO group).

Group IV received a single intra-tracheal instillation of bleomycin (4 mg/kg bw) and a daily intraperitoneal injection of RO (75 mg/kg bw) that started from the third day after fibrosis induction and lasted for 4 weeks (BLM/RO1 group, curative).

Group V received a daily intraperitoneal injection of RO (75 mg/kg bw) 15 days before the intra-tracheal injection of BLM and continued for 2 weeks after (BLM/RO2 group, prophylactic). All animals were sacrificed after 4 weeks of experimental treatments.

### Sample collection and analytical procedures

At the end of the treatment, rats were sacrificed after being anesthetized with urethane (40 mg/kg). Rats in all groups were killed by bleeding after incision of abdominal aorta. The left lung was fixed for the preparation of histological sections. The right lung was exploited for the measurement of indicators of oxidative stress after being weighted, homogenized in phosphate buffer saline and centrifuged. The supernatant was stored at −80 °C for eventual assays. Total soluble proteins level was evaluated according to the Biuret method previously described by Ohnishi and Barr (1978). It is based on the formation of a complex between soluble proteins and copper at acidic pH, and the absorbance is measured at 546 nm.

### Body weight determination

The body weight of rats was measured at the beginning and at the end of the experiment. The percentage of body weight gain in each group was compared and calculated by the following equation: [(final weight − initial weight)/initial weight] × 100.

### Histological study

For histological investigation, rat lungs were perfused and immersed in the fixative solution (10% neutral-buffered formalin) for 3 days, deposed in formalin, dehydrated through graded series of ethanol bath, embedded in paraffin, cut into 4 μm thick sections and finally stained with hematoxylin-eosin (H & E) to identify the inflammatory cells and with Masson’s trichrome to examine the deposition of collagen in different sections. In order to assess the severity and the extension of pulmonary lesion, we used a blinded semi-quantitative scoring system to evaluate the level of fibrosis in lung parenchyma. The severity of inflammation was estimated using the semi-quantitative grading system described by Ashcroft et al. (1988) to evaluate fibrosis progression in lung interstitium, which considers the following grades: Grade 0 = ‘normal lung’, Grade 1 = ‘minimal fibrous thickening of alveolar or bronchial walls’, Grades 2–3 =‘moderate thickening of walls without obvious damage to lung architecture’, Grades 4–5 = ‘increased fibrosis with definite damage to lung architecture and formation of fibrous bands or small fibrous mass’, Grades 6–7 = ‘severe distortion of structure and large fibrous areas’, ‘honeycomb lung’ was placed in this category; Grade 8 = ‘total fibrotic obliteration of the field’.

### Lipid peroxidation

Lipid peroxidation was determined according to the method of Ohkawa et al. (1979), which is based on the detection of thiobarbituric acid (TBA) reactive products. Reaction with TBA can detect small amounts of lipid peroxides, and more particularly the free malondialdehyde (MDA) produced during the oxidative breakdown of lipids and polyunsaturated fatty acids. Briefly, we incubated both lung supernatant and sodium phosphate buffer at 37 °C for 1 h and the mixture was centrifuged after being precipitated with 10% TCA (trichloroacetic acid). Then, we added 1% TBA to the supernatant and placed the mixture in the boiling water for 15 min. The absorbance was read at 532 nm and expressed in nmol/mg protein using molar extinction coefficient of 156,000 M ^−^ ^1 ^cm ^−^ ^1^.

### Glutathione *S*-transferase

Glutathione *S*-transferase (GST) activity was assayed using the method of Habig et al. (1974). In this experience, S-2,4-dinitrophenyl glutathione (CDNB) chosen as a substrate is conjugated with reduced glutathione and the formation of this association is proportional to the increase in absorbance at 340 nm against the blank. GST activity is expressed in μmol of CDNB-GSH conjugate formed/min/mg of protein.

### Catalase

Catalase activity (CAT) was determined using the method of Bonaventura et al. (1972) at 240 nm. Briefly, the reaction mixture included H_2_O_2_ in 0.019 M, 0.05 M phosphate buffer (pH =7) and 0.03 mL lung extract. CAT activity was calculated in terms of μmole of H_2_O_2_ consumed/minute/mg of protein.

### Thiols groups

Thiols groups are involved in the reactions of detoxification in organs. According to Genot (1996), this assay utilizes the DTNB (5,5-dithiobis-2-nitrobenzoic acid) in a redox reaction. At basic pH, free thiol (SH) of the protein, reduces the DTNB disulphide to yellow-u thiolate anion TNB [5-thio acid(2-nitrobenzoic acid)]. Lung supernatant (0.05 mL) is added to 1 mL of phosphate buffer, and the absorbance (*λ*_1_) was immediately determined at 412 nm. Then, 0.02 mL of DTNB was added and incubated for 15 min at room temperature to perform a second measure of the absorbance (*λ*_2_). A background tube containing only DTNB solution is measured in the same conditions. Calculation of the thiols group content was performed using following equation:

$$\mathrm{Thiol}\;\mathrm{groups}\;\left[\mathrm{mM}\right]=\left(\lambda_{2}-\lambda_{1}\right)\times 1.57$$

### Statistical analysis

For all *post-hoc* multiple comparisons, statistical significance was determined by ANOVA followed by Sidak’s test. A *p*-value of 0.05 or less was considered as statistically significant.

## Results

### Quantification of the ethanol extract constituents of *Rosmarinus officinalis*

**Figure 1.**
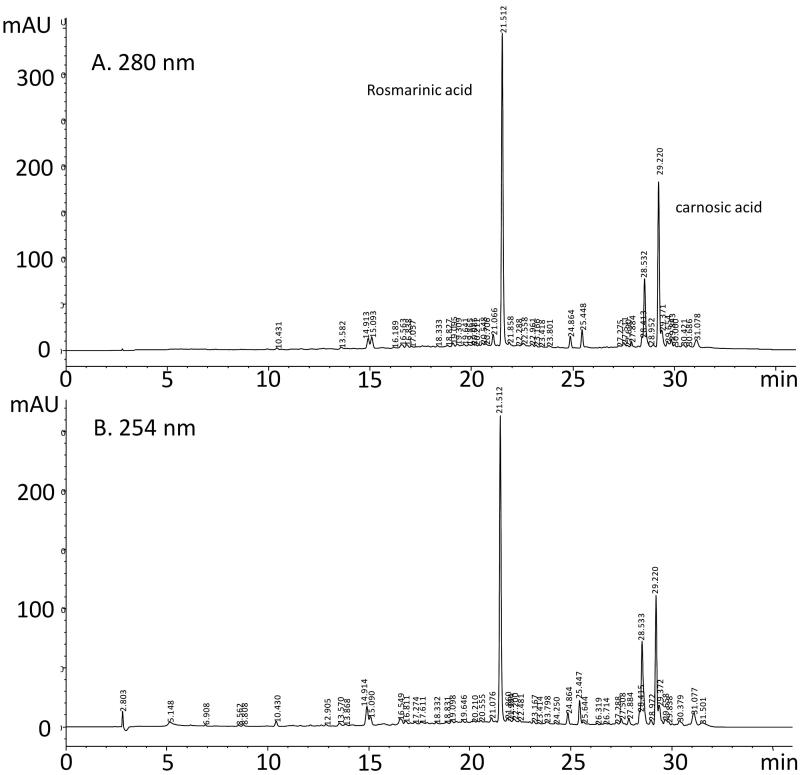
Chromatographic profiles of the ethanolic extract of rosemary recorded at two UV wavelengths: (a) 280 nm, (b) 254 nm.

Quantification of total polyphenols was determined by the Folin–Ciocalteu assay based on the colour intensity of the extract obtained. The analysis of the results (Table 1) shows that the aerial parts of rosemary contain high amounts of total polyphenols in the order of 60.52 mg GAE/g dw (mg equivalent gallic acid). This ethanol extract has also high levels of flavonoids in the range of 70.18 mg CE/g dw (mg equivalent catechin), but the levels of the condensed tannins are very low and do not exceed 0.3 mg CE/g dw. Our results (Figure 1) show that major constituents of the ethanol extract of rosemary are carnosic acid (tR =29.220 min, 6.886 mg/g dw) and rosmarinic acid (tR =21.512 min, 2.351 mg/g dw). This analysis indicates that carnosic acid is the major compound of this extract.

**Table 1.** Total polyphenol contents (TPC) expressed in mg equivalent gallic acid (GAE.g-1 DW), total flavonoid contents (TFC), condensed tannin contents (CTC) expressed in mg equivalent catechin (CE/g DW) and antioxidant activity of *Rosmarinus officinalis* leaves extract against DPPH radical expressed in IC_50_ value and iron reducing power (FRAP) expressed in EC_50_ value.

TPC (mg GAE/g dw)	60.52
TFC (mg CE/g dw)	70.18
CTC (mg CE.g-1 DW)	0.27
DPPH (IC_50_ μg/ml)	2.23 ± 0.14
FRAP (EC_50_ mg/ml)	74.13 ± 0.1

### Antioxidant activity of *Rosmarinus officinalis*

The reducing power assay was performed to show the ability of the extract to scavenge free radicals. The assay is based on the capacity of free radicals to reduce iron and DPPH. On the other hand, stabilization of DPPH may occur if it captures an electron or hydrogen. Thus, the antioxidant effect is proportional to the disappearance of DPPH^•^ in test samples. Our results show that rosemary ethanol extract reduces DPPH activity by half at 2.23 μg/mL. The scavenging effect of RO and BHT (butylated hydroxytoluene used as a standard in the DPPH assay) expressed as IC_50_ values are in order of 2.23 and 11.5 μg/mL, respectively, which demonstrates that *Rosmarinus officinalis* also has a better free radical scavenging activity than positive control (BHT). Our results also show that the rosemary extract has a better reducing activity of the ferric iron than ascorbic acid taken as a positive control (0.074 vs 37.33 μg/mL, respectively).

### Effect of rosemary extract on bleomycin-induced lipid peroxidation in lung

**Figure 2.**
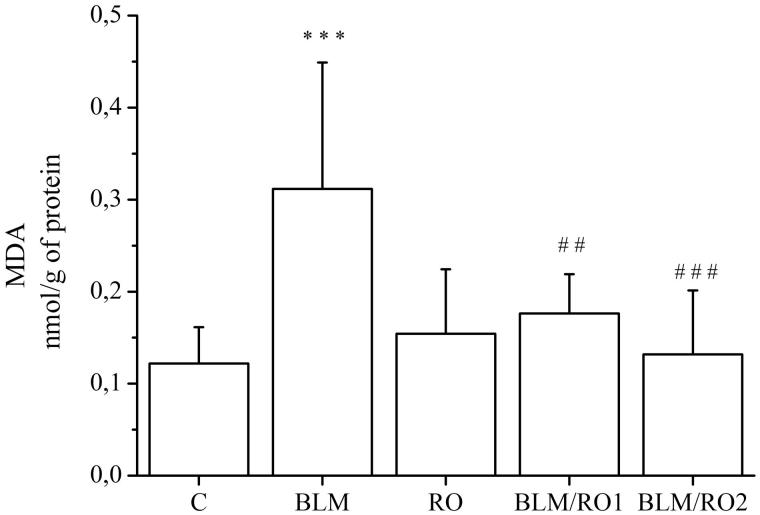
Effect of rosemary extract (RO) on bleomycin-induced lipid peroxidation in lung. Results are expressed as means ± S.D. (*n* = 10), ***p < 0.001 vs C*, ##p <* 0.01 vs BLM*, ###p <* 0.001 vs BLM.

Lipid peroxidation was assessed by MDA determination and the results are shown in Figure 2. MDA lung level is significantly higher in the BLM group compared to the control group (0.31 ± 0.13 vs 0.12 ± 0.03 nmol/g protein, respectively, *p* < 0.001). In the BLM/RO1 group (curative group), the MDA content is remarkably lower than those in BLM group (0.17 ± 0.04 vs 0.31 ± 0.13 nmol/g protein, respectively, *p* = 0.01). Similar result is also found in the BLM/RO2 group (prophylactic group), with a significant decrease in the MDA level when compared to the BLM group (0.13 ± 0.06 vs 0.31 ± 0.13 nmol/g protein, respectively, *p* < 0.001).

### Effect of rosemary extract on bleomycin-induced changes in catalase activity in lung

**Figure 3.**
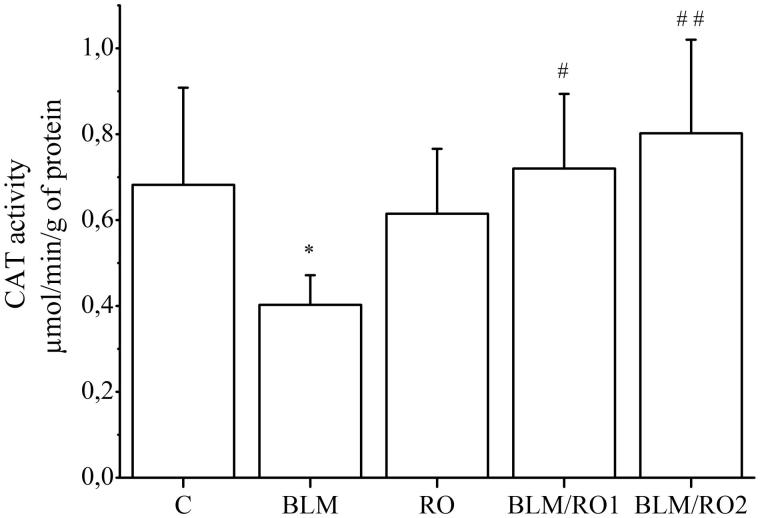
Effect of rosemary extract (RO) on bleomycin-induced changes in catalase activity in lung. Results are expressed as means ± S.D. (*n* = 10), *p < 0.05 vs C*, #p <* 0.05 vs BLM*, ##p <* 0.01 vs BLM.

The activity of catalase in the lung tissue, as shown in Figure 3, was significantly reduced by BLM treatment when compared with control (0.40 ± 0.06 vs 0.68 ± 0.20 μmol/min/g protein, respectively, *p* = 0.05), while in BLM/RO1 and BLM/RO2 groups, this activity is significantly restored when compared to the BLM group (0.70 ± 0.10 vs 0.40 ± 0.06 μmol/min/g protein *p* = 0.02 and 0.80 ± 0.20 vs 0.4 ± 0.06 μmol/min/g protein, *p* = 0.002, respectively).

### Effect of rosemary extract on bleomycin-induced changes in glutathione-*S*-transferase and thiols group levels in lung

**Figure 4.**
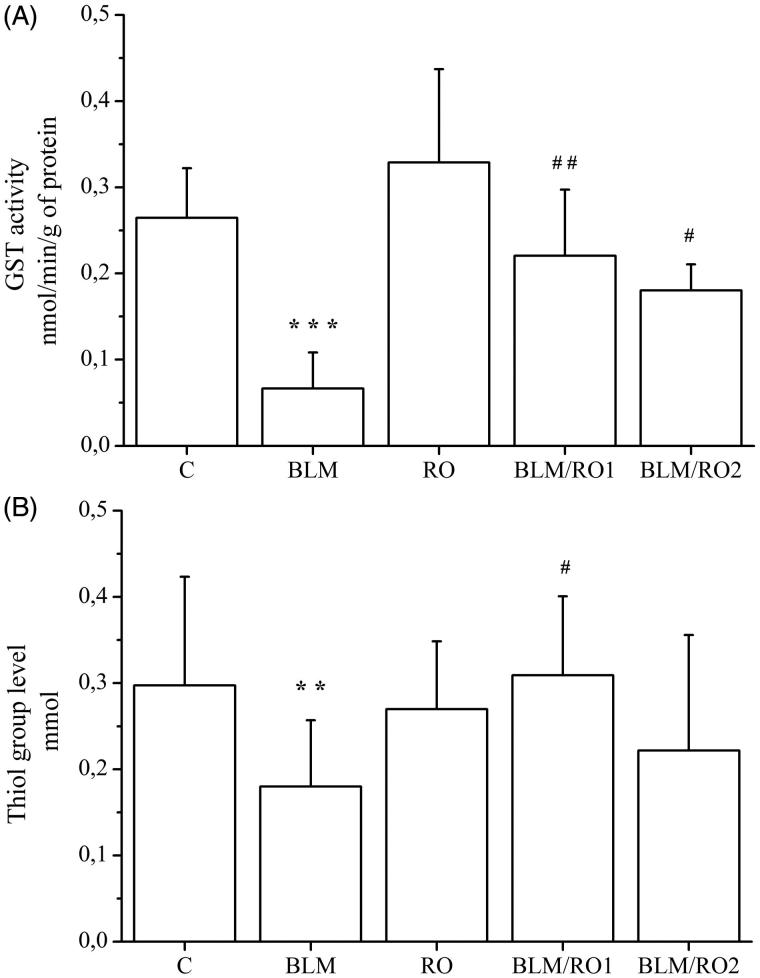
Effect of rosemary extract (RO) on bleomycin-induced changes in glutathione-*S*-transferase and thiols group levels in lung. Results are expressed as means ± S.D. (*n* = 10), ***p < 0.001 vs C, **p < 0.01 vs. C*, ##p* < 0.01 vs BLM, *#p* < 0.05 vs. BLM.

The level of GST content in lung tissue was significantly decreased by BLM treatment over control group (0.06 ± 0.04 vs 0.26 ± 0.05 nmol/min/g protein, respectively, *p* < 0.001) as shown in Figure 4(A), and this level was significantly increased in BLM/RO1 and BLM/RO2 groups when compared to the BLM group (0.22 ± 0.07 vs 0.06 ± 0.04 nmol/min/g protein, *p* = 0.006 and 0.18 ± 0.03 vs 0.06 ± 0.04 nmol/min/g protein, *p* = 0.05, respectively). Similarly, thiols levels were significantly reduced by BLM compared to control (0.16 ± 0.06 vs 0.34 ± 0.10 mmol, *p* = 0.01, respectively), and significantly increased in the BLM/RO1 group compared to the BLM group (0.3 ± 0.09 vs 0.16 ± 0.06 mmol, *p* = 0.04, respectively), but it did not reach a significantly higher level in the BLM/RO2 group when compared to the BLM group (0.22 ± 0.13 vs 0.16 ± 0.06 mmol, *p* = 0.9).

### Body weight variations

Death or clinical signs of rosemary extract poisoning were not detected in any of the experimental groups during this experiment. Body weight variations of rats are given in Table 2. We noticed a decrease in the body weight in the BLM group from the first to the last day before the sacrifice, but an increase in control, RO and BLM/RO2 groups in the same period with no significant change in the body weight in BLM/RO1 group after RO administration for 4 weeks. Our results showed a significant reduction of the percentage of body weight gain in the BLM group compared to control (−10.52 ± 4.64 vs 6.80 ± 2.76, *p* = 0.008) and a significant increase in this percentage in BLM/RO2 group compared to the BLM group (15.93 ± 4.25 vs −10.52 ± 4.64, *p* < 0.001, respectively).

**Table 2.** Body weight variations.

Body weight	C	BLM	RO	BLM/RO1	BLM/RO2
Initial (g)	195.5 ± 4.11	183 ± 5.06	200.8 ± 6.75	190 ± 6.10	223.6 ± 11.94
Final (g)	208 ± 3.59	162.5 ± 7.04	210 ± 3.57	190 ± 5.37	258 ± 14.45
Variation of weight (%)	6.8 ± 2.76	−10.52 ± 4.64^a^	6.92 ± 2.68	0.25 ± 2.15	15.93 ± 4.25^c^

Results are expressed as means ± S.D. (*n* = 10),^a^ *p* < 0.01 vs C, ^b^ *p* < 0.05 vs BLM, ^c^ *p* < 0.001 vs BLM.

### Fibrosis score

The gravity of fibrosis was estimated through the use the semi quantitative grading system and results are presented in Table 3. Fibrosis score is negligible in the RO group compared to the BLM group (0.60 ± 0.16 vs 3.7 ± 0.3, *p* < 0.001) while both BLM/RO1 and BLM/RO2 groups revealed a significant reduction in fibrosis score compared to the BLM group (2.33 ± 0.33 and 1.8 ± 0.32 vs 3.7 ± 0.3, *p* < 0.001, respectively).

**Table 3.** Fibrosis score.

Groups	Fibrosis score
Control	0.0 ± 0.0
BLM	3.7 ± 0.3
RO	0.6 ± 0.16^a^
BLM/RO1	2.33 ± 0.33^b^
BLM/RO2	1.8 ± 0.32^a^

Results are expressed as means ± S.D. (*n* = 10), ^a^ *p* < 0.001 vs. BLM; ^b^ *p* < 0.01 vs. BLM.

### Histopathological findings

**Figure 5.**
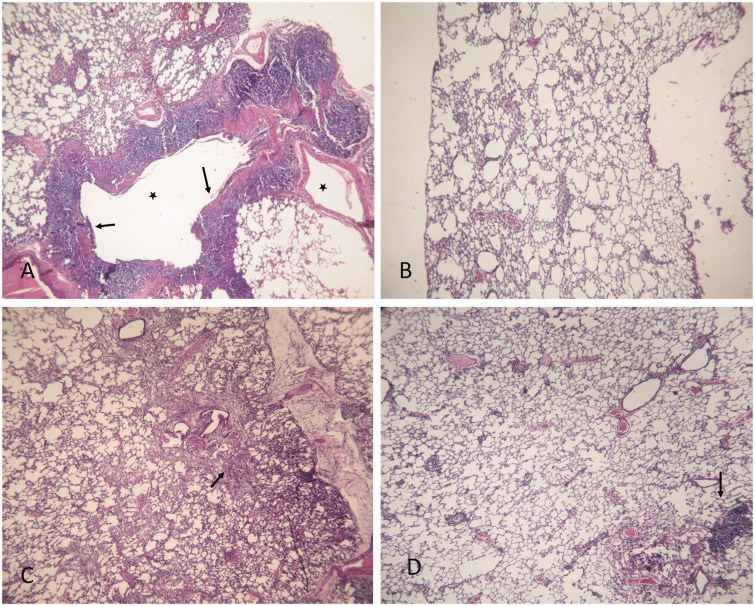
Histopathological findings (H&E) × 100 in lung tissue. (A) BLM, arrows indicate a thickening of interalveolar septum and formation of fibrous mass, asterisk indicates severe inflammation with the presence of large follicles which replace the parenchyma; (B) RO; (C) BLM/RO1, arrows point to a considerable interstitial thickening, but not that of alveolar walls; (D) BLM/RO2, arrows indicate slightly thickened walls without an apparent impairment to lung architecture. One representative example is shown for each group.

**Figure 6.**
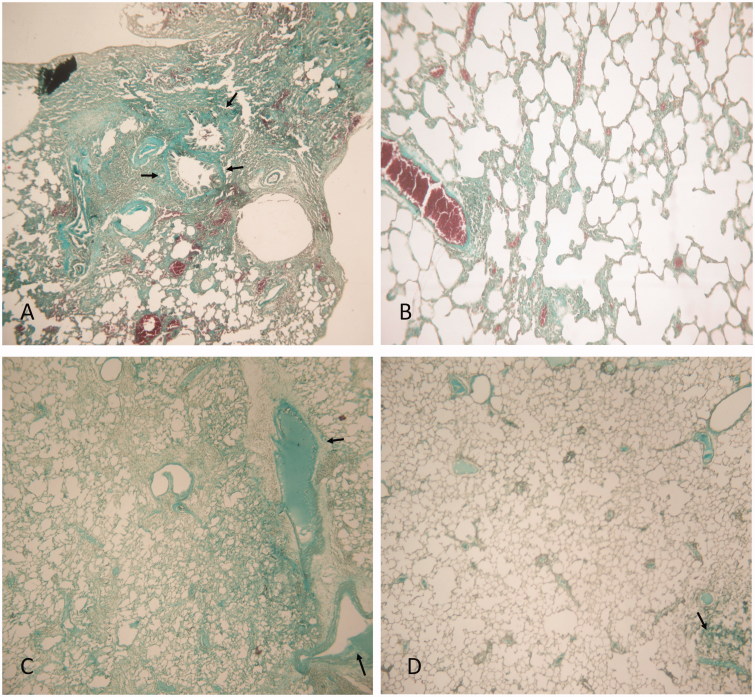
Histopathological findings (Masson’s trichrome) × 100 in lung tissue. BLM (A), RO (B), BLM/RO1 (C), BLM/RO2 (D). Arrows indicate condensed bundles of collagen. One representative example is shown for each group.

Figure 5 displays histopathological findings in lung tissue stained with hematoxylin–eosin. As expected, lungs from rats treated with BLM alone showed a thickening of interalveolar septum and formation of fibrous mass (Figure 5(A)). These animals also present a severe lung inflammation with the presence of large follicles, which replace the parenchyma (Figure 5(A)). Treatment with rosemary extract alone did not compromise normal lung architecture (Figure 5(B)). Lungs from rats of BLM/RO1 group (curative RO treatment) show a considerable interstitial thickening and near normal thickness of the alveolar walls (Figure 5(C)). Animals from prophylactic RO treatment group (BLM/RO2) present slightly thickened alveolar walls without an apparent impairment to lung architecture (Figure 5(D)). Figure 6 displays histopathological findings in lung tissue stained with Masson’s trichrome. Condensed bundles of collagen recognized in green colour are markedly evident in the BLM group (Figure 6(A)). Significant decrease in collagen deposition is observed in BLM/RO1 and BLM/RO2 groups of rats (Figure 6(C,D), respectively), while in RO group, normal levels of collagen deposition were detected (Figure 6(B)).

## Discussion

We aimed to investigate the effects of *Rosmarinus officinalis* leaves extract on lung fibrosis induced by BLM in adult Wistar rats in the present study. Since herbal medicine is based on plants consumption for therapeutic use and the effectiveness of these plants comes from their varied compounds and their different active ingredients, we have first determined the chemical composition of the extract, its phenol composition and its antioxidant activity. We have then evaluated its effects on the histopathological lesions and the oxidative stress induced by BLM in injured rat lung tissue.

Generally, the bioactivities of plant extracts are often linked to their phytochemical composition like polyphenols, flavonoids and condensed tannins. Our results show that rosemary extract has a high amount of total polyphenols and a better reducing activity of the ferric iron than the ascorbic acid taken as a positive control. Moreover, RO has a better free radical scavenging activity than the positive control (BHT). This antiradical activity reflects the high-antioxidant potential of this plant, which may however vary significantly according to the part used, herb provenance and the solvent of extraction. Phenolic compounds include a conjugated ring with an hydroxyl group and can react like an antioxidant inhibiting the progression of diseases related to free radicals (Robak & Dryglewski 1988). The antioxidant capacity in plants is characterized by quenching free radicals, and plant extracts have scavenging abilities that force free radicals in a complex assay system to eliminate several pathological troubles (Adedapo et al. 2008).

In the human body, antioxidant rates must be in equilibrium with the level of reactive oxygen species to prevent oxidative stress, and in our study, the levels of some oxidative stress indicators were evaluated. According to Sausville et al. (1978), the cytotoxicity of BLM may be explained by the production of free radicals. BLM forms a complex with Fe (II), which is posteriorly oxidized to Fe (III), causing a reduction of oxygen to free radicals. Our results show that BLM caused an increase of MDA level in the lung. The MDA is formed upon decomposition of the polyunsaturated fatty acids on exposure to free radicals. The increased level of MDA in lung can be explained by the intracellular formation of ROS by BLM that attacks cell membranes and organelles causing a misbalance between the formation of reactive oxygen species and the capacity to scavenge them in a cell. On the other hand, the addition of RO in both curative and prophylactic treatment caused a significant decrease of MDA level. This result supports the hypothesis that RO can neutralize oxidative stress, scavenge free radicals and protect the cell from their toxicity that lead to the DNA damage and cell death and accordingly to lung injury.

The level of GSH (reduced glutathione) in the body is intimately linked to the activity of several enzymes, such as GPx (glutathione peroxidase), glutathione reductase and GST (glutathione-*S*-transferase). Glucose 6-P dehydrogenase and glutathione-*S*-transferase are able to decrease the level of peroxide and preserve a steady supply of metabolic intermediates like NADPH and GSH that are important for a best activation of primary antioxidant enzymes and help in ROS detoxification (Vendemiale et al. 1999). Consistent with oxidant production and lipid peroxidation, GST and thiol group levels were significantly decreased after bleomycin administration. According to Venkatesan et al. (1997) and Cortijo et al. (2001), GSH is necessary for the protection of thiols and other nucleophilic groups of proteins from free oxygen radicals. Antioxidants such as catalase, superoxide dismutase and GST are protecting enzymes and have the ability to remove toxic insults derived from chemicals, oxidative stress and metabolites from cells (Singh et al. 2002). The addition of RO in both curative and preventive treatment caused a significant increase of GST and thiol group levels in the lung.

An important feature of BLM-induced pulmonary fibrosis in rats is that, activated phagocytes also generate a big quantity of reactive oxygen species after bleomycin administration. The released ROS comprise hydroxyl radicals, superoxide anion, hydrogen peroxide and nitric oxide (Gao et al. 2008). Catalase plays an essential role in the maintenance of hydrogen peroxide homeostasis in cells, since it can convert 6 million molecules of hydrogen peroxide to water and oxygen per minute (Valko et al. 2006). Our results show that BLM induced a significant decrease in catalase activity in lung. Odajima et al. (2010) demonstrated that, contrarily to the evolution of human pulmonary fibrosis, reduction in catalase activity is transient, because catalase levels seemed to rise again after 21 days and tend to be re-established 35 days before the resolution phase, suggesting that the change in cellular dynamics during fibrosis progression is responsible of the variation in the level of catalase in bleomycin-induced lung fibrosis. The fact that the catalase activity in BLM/RO1 and BLM/RO2 groups is significantly restored when compared to the BLM group could indicate that RO might modulate cellular dynamics in fibrotic processes.

In conjunction with the above suggestion, our results obtained from curative treatment group of animals indicate that rosemary extract exerts a restoring effect on BLM-induced lung structural disorganization. Not only this suggests that both oxidant and fibrotic responses are linked but also predicates that the extract might have a cytostatic and/or cytotoxic effect on the cells that are abnormally activated in this lung fibrosis model.

Our rosemary extract contains a large quantity of carnosic acid and rosmarinic acid, which are both antioxidants (Sánchez-Campillo et al. 2009; Zang et al. 2015). But with the present results it is difficult to pin-point, which one of these molecules has the major anti-fibrotic effect, or whether is it a result of their synergistic contribution. In fact, rosmarinic acid was first reported to have an antifibrotic effect on experimental liver fibrosis by the inhibition of hepatic stellate cells proliferation, TGF-beta1 and CTGF expression in these cells, and in CCl_4_-induced liver fibrosis in rats (Li et al. 2010). Rosmarinic acid treatment in mice with existing cholestatic liver fibrosis was also reported to inhibit hepatic stellate cells activation, known by the induction of myofibroblast activation and the progression of liver fibrosis (Yang et al. 2012). On the other hand, carnosic acid can suppress the increase of metalloproteinase expression in human dermal fibroblasts exposed to UV radiation (Offord et al. 2002; Park et al. 2013) and can reduce the increase in ROS level induced by UV, which reflects both antioxidant and antifibrotic activities (Park et al. 2013). Carnosic acid can also exert an anti-inflammatory action in skin by attenuating several interleukins and chemoattractant protein production, following an inflammatory reaction (Oh et al. 2012). Secondly, carnosic acid may have medicinal applications for the treatment of many forms of cancers. This herbal molecule can induce HepG2 cell death via Akt inhibition, implicating both apoptosis and autophagy in these cells (Xiang et al. 2013; Gao et al. 2016) or only apoptosis via caspase activation in human renal carcinoma caki cells (Min et al. 2014), and in cultured colorectal cancer cells (Barni et al. 2012). Thus, we can speculate that carnosic acid can play an antifibrotic role by the induction of activated fibroblasts apoptosis, because the accumulation of these cells is considered as a potential cause of several forms of fibrosis.

Carnosic acid has also been described to cooperate with other natural compounds to produce a synergistic anti-proliferative effect in human myeloid leukemia cells, reflected in an important apoptotic cell death. These compounds include 1,25-dihydroxyvitamin D3, all-*trans* retinoic acid (Steiner et al. 2001; Ren et al. 2008) and curcumin (Pesakhov et al. 2016). It is, thus, very plausible that carnosic acid cooperated with other phenol compounds in our rosemary extract, rosmarinic acid, for example, to boost its antifibrotic and antioxidant activities.

## Conclusions

Considered together, our results demonstrate that *Rosmarinus officinalis* leaves extract can attenuate bleomycin-induced lung fibrosis by normalizing pro-oxidant parameters and by enhancing the activities of antioxidant enzymes. Indirectly, these results suggest that the constituents of the extract may modulate cellular dynamics during fibrosis progression, but further studies need to be done to elucidate the molecular mechanisms of action of this whole plant extract as well as of each of its phenol compounds, both *in vivo* and *in vitro*.
